# Angiofluorographic aspects in age-related macular degeneration

**Published:** 2014

**Authors:** A Tomi, I Marin

**Affiliations:** * Department of Ophthalmology, “Carol Davila” University of Medicine and Pharmacy, Bucharest, Romania; ** Clinical Hospital for Ophthalmic Emergencies, Bucharest, Romania

**Keywords:** age-related macular degeneration, fluorescein angiography, classification, non-exudative and exudative patterns

## Abstract

Although AMD (age-related macular degeneration) has been described for over 100 years, there is neither a standard agreement on the definition of specific lesions nor a generally accepted classification system. For example, the age limits for AMD varied widely in different clinical studies; the methods used for examination also vary (visual acuity, perimetry, contrast sensitivity, slit lamp examination of the fundus, retinal photography, fluorescein angiography, indocyanine green angiography).

We described the multitude of angiofluorographic aspects in patients with AMD and conceived a classification to be easily used in clinical practice.

Although a detailed ophthalmoscopy can often identify the characteristic lesions of AMD, a complete picture is obtained by fluorescein angiography. The angiographic classification of AMD is structured similarly to the clinical one. It has two main patterns, non-exudative and exudative lesions, but it provides more information about the nature of the lesions.

In the last three decades, an impressive amount of information regarding the prevalence, progression and risk factors for AMD has been published. The source of this information is mainly represented by the large population studies that are often multicenter studies. Recognizing the clinical signs of AMD and classifying them into different stages is important for the prognosis and the therapeutical decision, but also for conceiving study protocols.

## Introduction

In 1855, Donders noted for the first time the “lumpy” findings called *drusen*, and in 1875, the disciform macular lesion was described. Otto Haab is the one who observed an association between the clinical aspect of atrophic pigmentary changes in the elderly patients with central visual dysfunction, a condition he named *senile macular degeneration* (1885) [**1**]. In 1967, Gass brought up the hypothesis that drusen, atrophic AMD and disciform AMD are clinical aspects of the same disease [**2**].

Widely, AMD includes any degenerative macular process, atrophic or exudative, that appears at over 50 years of age and determines a decrease in the visual acuity, and also certain pigmentary macular lesions (for example: drusen) that predispose to these complications.

The definitions proposed by different group studies have known significant variations regarding the age limit and the staging of the disease.

In 1995, Bird, Bressler and Bressler proposed and published an international classification and aggrading system for AMD [**3**,**4**].

They defined AMD as a degenerative condition that appears in people of over 50 years old and it is characterized by the following macular pathologic changes (that are not the consequence of another condition, like trauma, retinal detachment, myopia, choroid inflammation or vascular retinal disease):

- soft drusen ≥ 63 µm

- hyper- and/ or hypopigmentation of the retinal pigmentary epithelium (RPE)

- RPE detachment (PED) ± neuroepithelium detachment

- geographic atrophy of the RPE

- fibrous subretinal scars

- retinal hemorrhages or hard exudates in the absence of other retinal (vascular) disease.

The visual acuity is not taken into consideration as a factor neither in the definition, nor in the classification.

The **incidence** of the disease is continuously growing as a consequence of the increasing number of the elderly population on one hand, and on the other, of the improvement of diagnostic methods. Scientific progress brought successful treatment for other ocular diseases that affect the elderly (cataract, glaucoma) and so AMD became the leading cause of blindness in patients over 65 years old [**1**,**6**,**7**].

The **prevalence** data varies in different studies and it is estimated around 6-10% in the 65-74 years age group and around 19-30% in the population aged over 75 [**1**,**5**].

The impact of AMD upon the society is continuously increasing, leading to a more intense clinical activity and research in the field.

## Methods

Starting from the MPS classification of AMD, we tried to describe all the angiographic aspects we have noticed in our patients and a classification was proposed.

**A. Non-exudative aspects in AMD**

**1) Drusen**

Although drusen are frequently observed in elderly patients (50% of the population over 70 years old), they are part of AMD and they can present various clinical and angiographic aspects. The drusen may vary in number, size, shape and location and can be associated with pigmentary changes.

Because certain characteristics of drusen have an important prognostic value, it is useful to recognize them clinically and on the angiogram and to describe their aspect [9]. Taking into account the size and appearance of the lesions, there are two main types of drusen: soft and hard.

**Hard drusen** result by the local accumulation of material under the basement membrane of the RPE associated with the thinning and loss of pigment in the overlaying epithelium. 

**Fig. 1:**
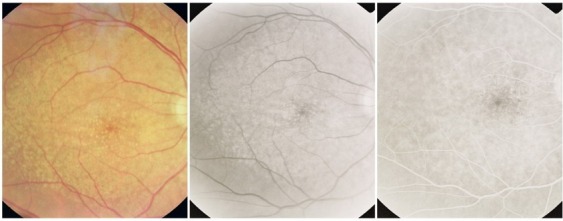
Hard drusen

On the ***angiogram***, they appear as multiple hyperfluorescent spots of small size, round shaped, well contoured, obvious from the early phase of the FA; their aspect remains constant throughout the FA, until the late phase, when they fade away together with the choroidal fluorescence. They represent small “window defects” of the overlaying RPE, which is thin and has lost its pigment.

**Fig. 2:**
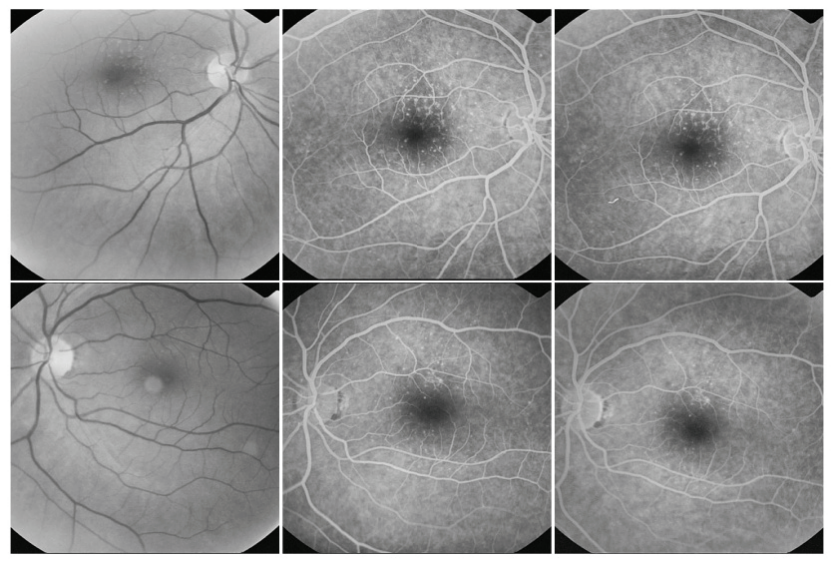
Perifoveal drusen

Their size is around 50-60µ. As for the distribution, they can be found in the macular region, along the vascular arcades (perivascular drusen) or more peripheral. 

**Fig. 3:**
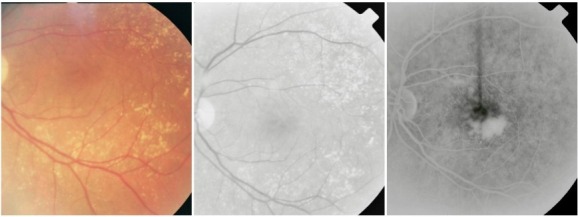
Perivascular drusen associated with CNV

They can vary in number: sometimes an isolated drusen can be observed, other times numerous drusen are found spread all over the posterior pole. The solitary drusen can be interpreted on the FA as “RPE defect”.

**Soft drusen** are larger in size than hard drusen (>60µ) and not so well contoured. They represent small RPE detachments and they can appear underneath the hard drusen ”swallowing” them. They form around the age of 50 or later and their presence is an indicator of high risk for advanced atrophic or exudative AMD. They tend to conflate.

On the ***angiogram***, soft drusen appear as round hyperfluorescent lesions, lager than hard drusen, with a poorly defined contour and the tendency to conflate. Their intensity is lower than that of hard drusen in the early phases. In the late phase, the hyperfluorescence does not expand beyond the limits observed in the early phases, but they do present pooling, which persists longer.

**Fig. 4:**
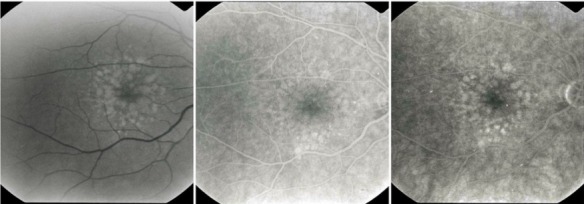
Soft drusen

It is very common for hard drusen to be found in association with soft drusen, and this clinical aspect was named **mixt drusen.**

**Fig. 5:**
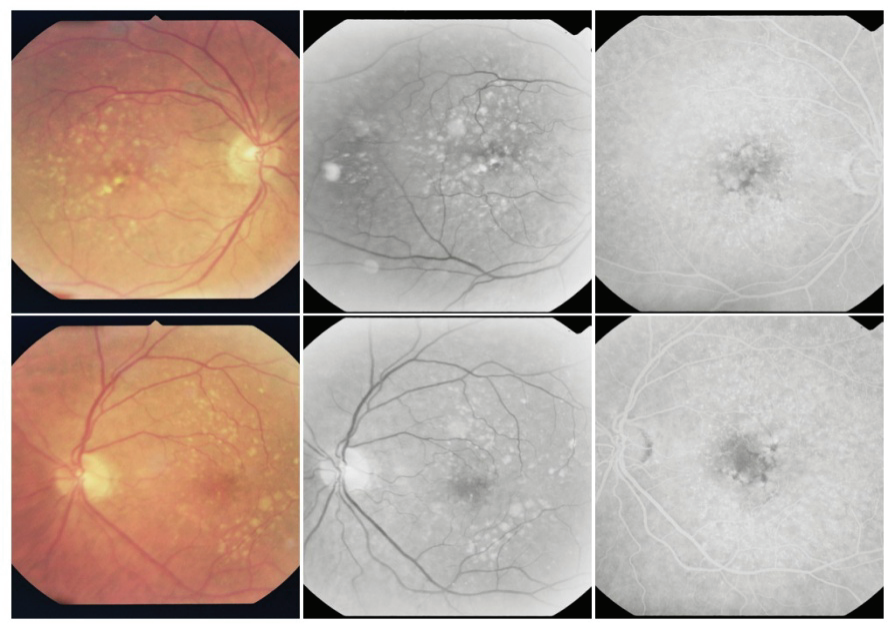
Mixt drusen

The tendency to confluence that soft drusen present is often observed in the perifoveal area, where the hyperfluorescent lesions begin to merge (**drusen with tendency to confluence**).

After having merged, **confluent drusen** can be interpreted as a serious detachment of RPE, the differential diagnosis between the two aspects being very difficult (**drusenoid PED**).

**Fig. 6:**
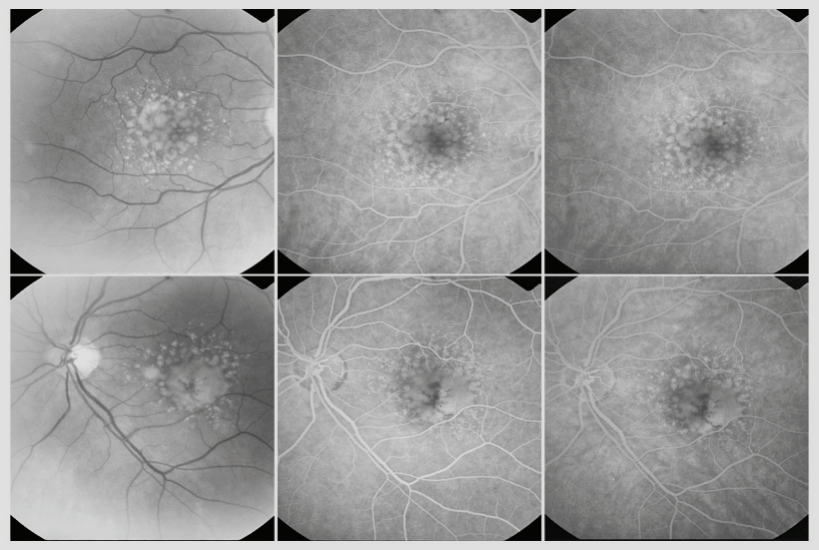
Drusen with tendency to confluence (OD), confluent drusen (OS)

Sometimes the angiogram reveals **special forms** of drusen. 

**Cuticular drusen** (basal laminar drusen) are a form of nodular, hard drusen, that appear as numerous, pin sized, yellowish, subretinal lesions, spread across the posterior pole and very discrete upon ophthalmoscopic examination.

On the ***angiogram***, they become obvious and present as multiple pin sized hyperfluorescent lesions, which tend to fade away in the late phases. Their aspect has been compared to “stars in the sky” or “the milky way”.

**Fig. 7:**
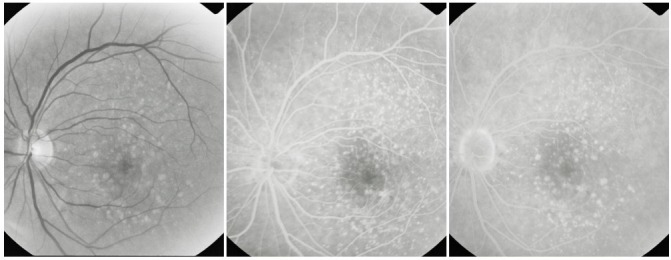
Cuticular drusen

Cuticular drusen can be found in young adults. As these patients age, these lesions can be associated with soft drusen, which indicates a high risk for progression of AMD.

**Pseudoreticular drusen** appear as a yellowish network with intrareticular spaces of approximately 125-250µ, present first superiorly to the macula, then expanding in a circumferential manner inferiorly to the macula and beyond the macular area [**10**].

**Fig. 8:**
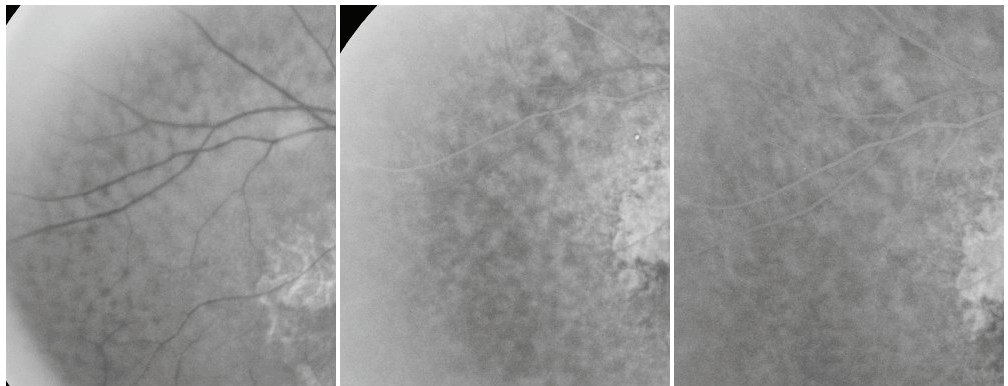
Pseudoreticular drusen

They are not always visible on the angiogram as they are not hyperfluorescent. The best way to detect them is by using red-free light or the He-Ne laser of the Heidelberg scanning laser ophthalmoscope.

They are considered a risk factor for exudative AMD [**10**].

Histologically, pseudoreticular drusen appear as a consequence of changes in the choroid. The small vessels that are normally found in the middle choroidal layers and between the large choroidal vessels disappear and this explains the network-like aspect.

**Fig. 9:**
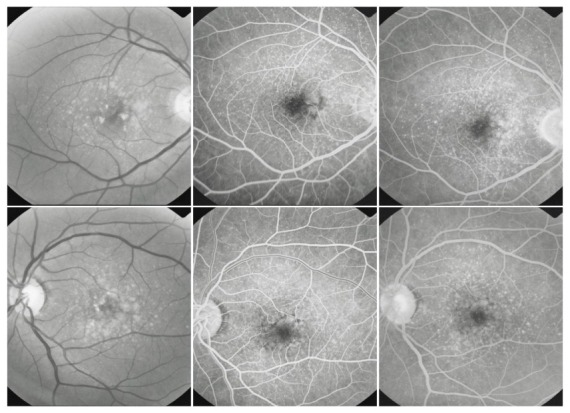
Drusen associated with alterations of the pigmentary epithelium

Both hard and soft drusen can be accompanied by pigmentary alterations, consisting of atrophy or hypotrophy of the surrounding RPE, due to the abnormal proliferative activity of the RPE (**drusen with alterations of the RPE**).

**2) Pigmentary epithelium alterations (non-geographic atrophy**)

 RPE alterations found in AMD associated or not with drusen have also been called **non-geographic atrophy** and they consist of pigment irregularities, hypo and hyperpigmentation, arranged sometimes in a reticular pattern.

On the ***angiogram***, the areas of non-geographic atrophy present hyperfluorescent spots that correspond to the atrophic areas and a reticular hypofluorescence corresponding to the pigment deposits in the RPE. The aspect is constant throughout the entire angiogram. 

**Fig. 10:**
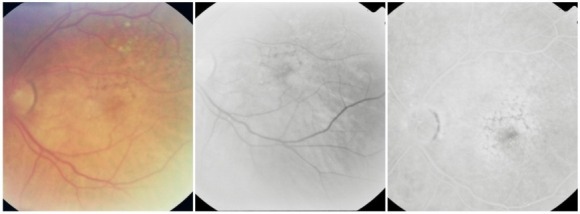
Non-geographic atrophy

**3) Geographic atrophy**

When RPE atrophy is associated with choriocapillary atrophy, the choroidal vessels become visible ophthalmoscopically and on the angiogram. In these particular areas, photoreceptors are also lost and that determines a decrease in visual acuity.

A photographic documentation of the evolution of lesions that leads to atrophy rarely exists, but several patterns of progression have been described [**12**,**13**]:

- Progression of the atrophy in areas with pigmentary alterations (primary geographic atrophy)

- Drusen regression

- Following the resorption of a serous PED 

The islands of geographic atrophy appear in the perifoveal region and tend to conflate and expand towards the fovea.

**Fig. 11:**
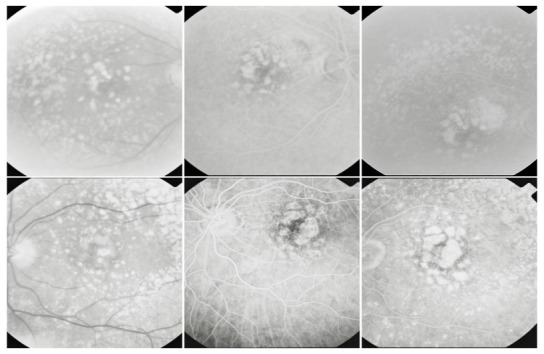
Atrophic areas that tend to conflate

On the ***fluorescein angiogram***, these areas appear hyperfluorescent, with good visibility of the choroidal vessels – an image similar to that of a geographic map. The hyperfluorescent area does not expand in the late phases beyond its well-defined initial contour.

**Fig. 12:**
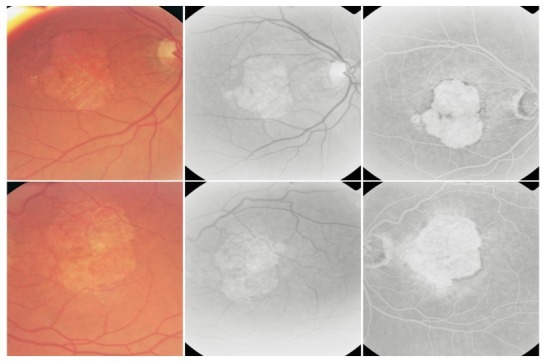
Central geographic atrophy

Most of the times, geographic atrophy is found bilaterally and the lesions tend to be symmetrical. The foveal “sparing” sometimes explains the relatively good visual acuity found in patients with large, central atrophy.

**Fig. 13:**
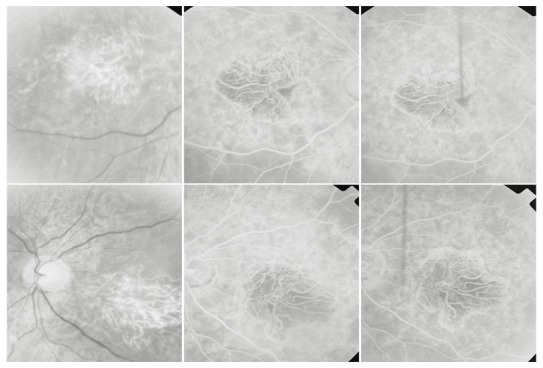
Geographic atrophy with foveal sparing in OD

**B. EXUDATIVE ASPECTS IN AMD**

When subepithelial or subretinal fluid is identified, the AMD lesion is classified as “wet”, neovascular or exudative and the impact on visual acuity is much more severe.

The MPS (Macular Photocoagulation Study) Group initiated the classification of neovascular AMD based on the angiographic aspect. They defined terms like classic CNV and occult CNV [**8**]. Back then, laser photocoagulation and photodynamic therapy were the available therapeutic options and it was imperative to correctly establish the type of lesion using the FA. The angiographic characteristics of the lesion influenced the therapeutical decision and determined whether a patient was eligible or not for a certain therapy.

The angiographic diagnosis of AMD aims to reveal the exudation (leakage of the dye) and its source. In dry AMD, the angiographic aspect is constant throughout the FA phases, but that is no longer the case for wet AMD. The neovascular form presents a dynamic change of aspect and draws attention towards the presence of a source of leakage, which is the choroidal neovascularization. It appears from the very beginning of the angiogram, when the choroidal flush determines a hyperfluorescence that will then grow in size and intensity, expanding beyond its initial contour. By contrast, the areas of atrophy which also present with early hyperfluorescence (by window defect) do not expand beyond their well defined initial limit [**11**,**18**]. 

In some cases, the source of leakage can be fully revealed and this is called a classic, well-defined choroidal neovascularization (CNV). In others, the source remains obscure and the only information obtained by angiogram is that leakage is present and that is called occult, undefined CNV. There are of course cases in which both the leakage and its source are “masked” by lesions that block the fluorescence (hemorrhage for example).

**1. Classic, well defined CNV**

It can be completely identified from the early phases, as it appears during the arterial phase. The aspect is that of an intense, well-defined hyperfluorescence; sometimes the capillary network of the neovascularization appears as “lacy” pattern.

In the late phase, the hyperfluorescence intensifies and expands beyond its initial limits. The contour becomes less well defined.

**Fig. 14:**
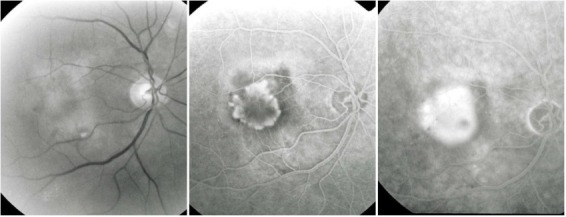
Classic CNV

**2. Occult CNV** can present 2 angiographic patterns:

a. **Type 1 occult CNV** or fibrovascular PED

It appears as an irregular hyperfluorescence, with poorly defined contour and a rounded shape. It becomes obvious of the angiogram later than classic CNV and it is more intense in the late phase.

**Fig. 15:**
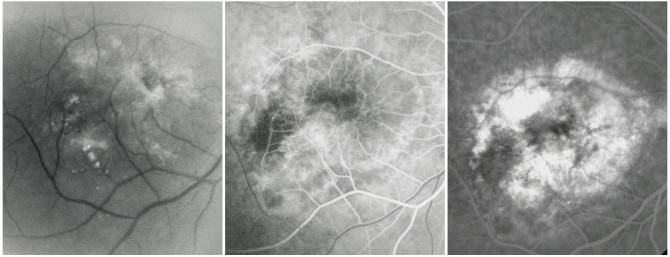
Type 1 occult CNV (fibrovascular RPE detachment)

b. **Type 2 occult CNV** or late leakage of an undetermined source (diffuse CNV, “diffuse ooze membrane”)

Characteristic are multiple hyperfluorescent lesions, pinsized or larger (spots), that become more intense in the late phase, presenting leakage. In the early phase, there is no sign of classic CNV or fibrovascular RPE detachment, and the leakage is diffuse. These diffuse membranes are poorly contoured and usually do not present intense exudation. The source of leakage cannot be identified in the early phase.

**Fig. 16:**
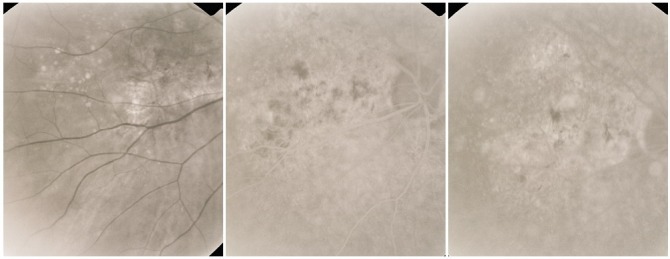
Type 2 occult CNV (diffuse exudation)

**3. Serous RPE detachment (PED)**

It may be produced by a CNV, but it is “masked’ by the intense hyperfluorescence of the detachment. A serous PED appears as an early, intense, uniform, well contoured, round shaped hyperfluorescence that maintains its aspect all through the angiogram.

**Fig. 17:**
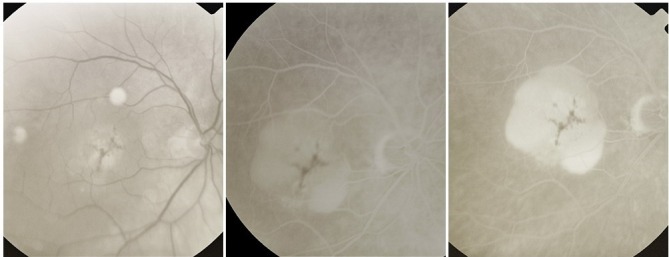
Serous PED

 Certain angiographic elements associated to the detachment may suggest the presence of an occult CNV.

I. A “notch” on the round shaped PED [**18**]

**Fig. 18:**
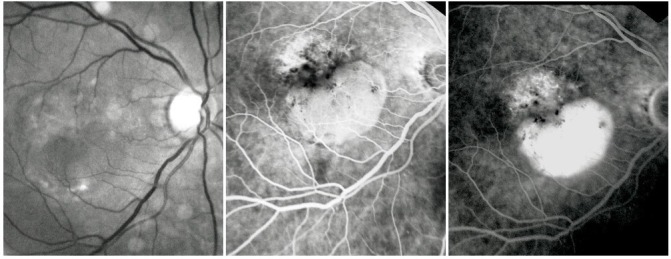
PED with “notch”

II. One or more hyperfluorescent spots obvious before the “filling” of the serous PED, the so-called “hot spots”.

**Fig. 19:**
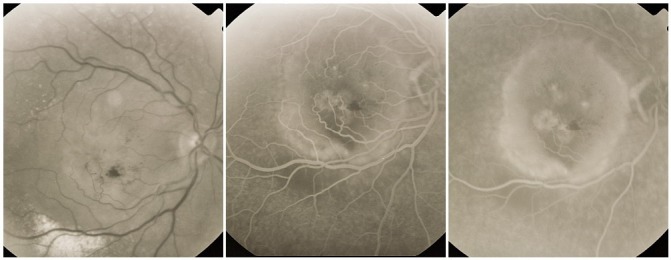
PED with “hot spots”

III. The association with hypofluorescent areas corresponding to the hemorrhagic blockage (**serous-hemorrhagic PED**)

**Fig. 20:**
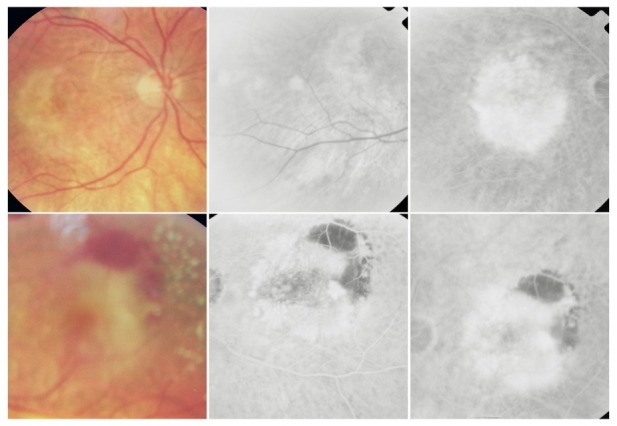
OD: Type 1 occult CNV, OS: Hemorrhagic PED

**4. Retinal angiomatous proliferation (RAP)**

Some occult angiographic aspects have been later explained by Harnett and co. [**14**], who in 1992, described for the first time, a special form of neovascular proliferation, originating from the retinal vessels and called **retinal angiomatous proliferation**. Later, Yannuzzi and co. (2001) [**15**] showed that this proliferation originating from the deep retinal layers expands creating a retinal-choroidal anastomosis.

Other authors (Gass, 2003) do not agree with the retinal origin of this neovascular complex and state that it has a choroidal origin, thus calling it ***occult chorio-retinal anastomosis***. Gass has also elaborated a 5-stage development scale. More recently (2008), Freund and co. [**16**], reanalyzed this issue and concluded that this vascular anomaly has a dual origin, retinal and/ or choroidal.

These vascular abnormalities are associated with PEDs, intraretinal fluid accumulation in the form of cysts and also with subretinal fluid (serous detachment of the neurosensory retina – SDNSR).

Clinically, it presents as small, multiple, intra and preretinal hemorrhages, numerous hard exudates and PED. The lesions are juxtafoveal and tend to be bilateral. Sometimes the anastomosis can be observed. They have a constant caliber (they do not get thinner like retinal vessels do) and are directed towards the deeper layers of the retina. Associated drusen and pigmentary changes may also be present [**18**].

The FA may be useful in detecting IRN and intraretinal vascular anomalies, but it has a limited value in the diagnosis, assessment and classification of RAP. The indocyanine green angiogram, which reveals the choroidal vessels, is a better tool in these cases. However, recognizing this particular form of AMD is important for the prognosis, because RAP progresses differently and responds differently to therapy compared to other CNV.

On the angiogram, RAP mimics the aspect of an occult or a minimally classic CNV, but the PED associated with it is not revealed. It may appear as a small early intense hyperfluorescence that presents diffuse leakage, determining a late, irregular diffuse hyperfluorescence. Usually, the vascular abnormality is situated at the tip of a retinal vessel.

**Fig. 21:**
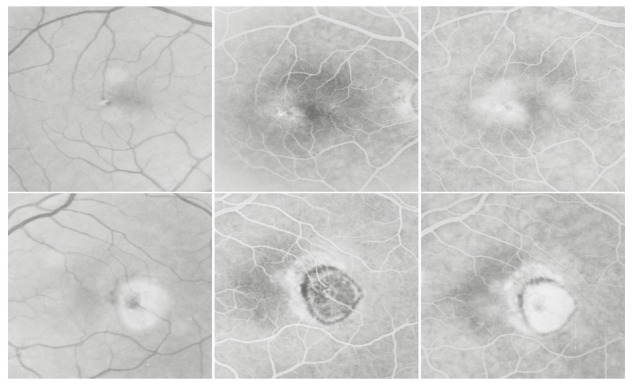
OD: RAP with vascular PED; OS: RAP with classic CNV

The particular aspect of the RPE detachment produced by RAP comes from the different origin of this vascular abnormality, which implies a different dynamic of exudation. In choroidal neovascularization, the leakage first fills the subepithelial space and then it appears intra- and subretinal. In RAP, the retinal leakage is primary and the RPE detachment appears later, and thus it is sometimes masked by the intra- and subretinal fluid accumulation.

**Fig. 22:**
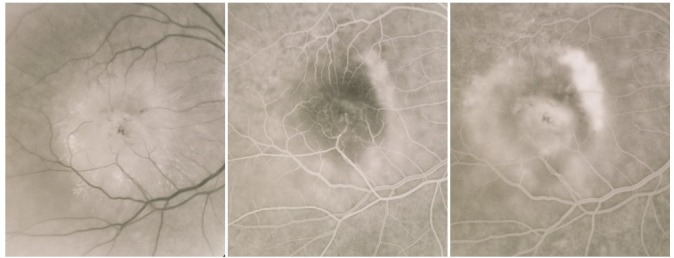
RAP with retinal-choroidal anastomosis

Nowadays, RAP is considered a distinct subtype of exudative AMD, with a prevalence around 8-15%, a tendency to manifest bilaterally and associated with a poor prognosis.

**5. Subretinal hemorrhage (CNV with subretinal hemorrhage)**

It determines a blockage of the fluorescence caused by CNV whether classic or occult. On the angiogram, a deep, compact hypofluorescent area on top of which the retinal vessels filled with dye can be observed. 

**Fig. 23:**
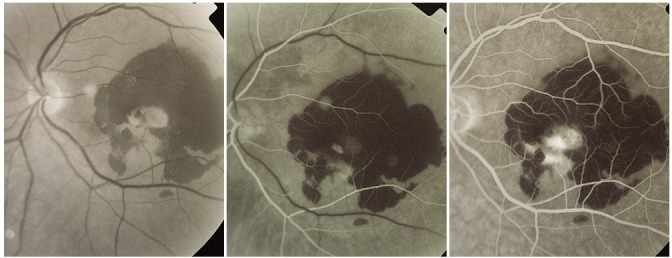
Predominantly occult CNV with subretinal hemorrhage

It is sometimes predominant, while other times it only partially masks the CNV or it appears as a hypofluorescence edge around the neovascular membrane or as a small hypofluorescence situated right next to it. There are situations in which CNV determines a massive subretinal hemorrhage. Rarely, CNV can produce a vitreal hemorrhage and in this situation, the visualization of the retina is reduced. 

**6. CNV with tear of the RPE**

The tear the RPE can appear spontaneously or after therapy (laser or intravitreal injection with anti-VEGF agents) and it usually determines a sudden important decrease in visual acuity [**17**].

On the FA, the aspect is characteristic and it consists of a highly hyperfluorescent area that appears from the early phases. It is well contoured, crescent shaped and it corresponds to the area no longer covered by RPE. On the concave edge of this hyperfluorescent crescent one can observe a hypofluorescence area, due to the blocking effect of the “rolled” RPE. Sometimes, in the late phases, in this area exudation can appear indicating the presence of underlying CNV.

**Fig. 24:**
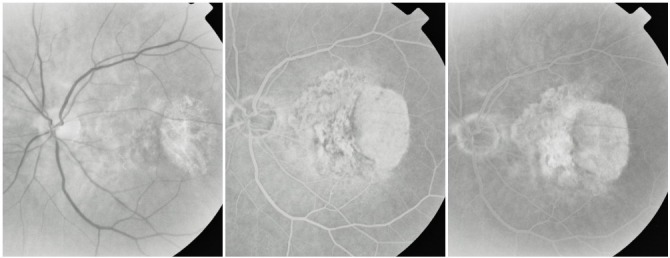
CNV with tear of the RPE

**7. Fibrovascular CNV**

In CNV that progresses towards scar tissue, the fibrous tissue tends to expand. The angiogram reveals in variable amounts both hyperfluorescent lesions that present exudation (leakage) and hyperfluorescent lesions which are non-exudative and present staining.

**Fig. 25:**
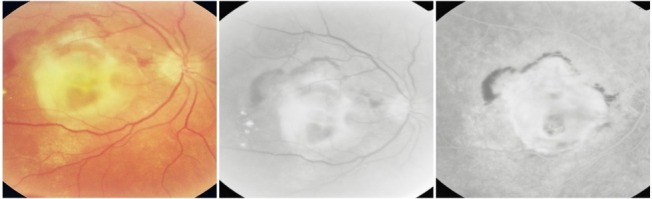
Fibrovascular CNV with subretinal hemorrhage

**8. Cicatricial (disciform) CNV**

When the fibrous lesion no longer presents exudation, the CNV has reached the cicatricial stage. The disciform scar rapidly stains, presenting an intense hyperfluorescence that remains well contoured and does not present leakage. Some lesions are noticed occasionally in the scar area:

- pigmented areas, visible both clinically and on the angiogram

**Fig. 26:**
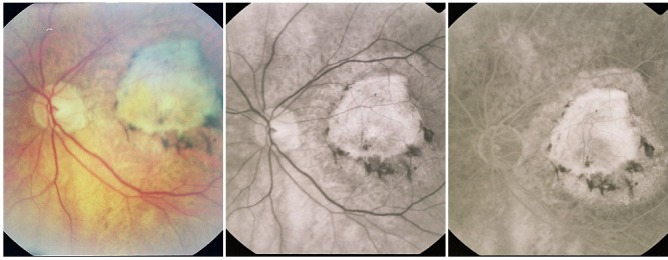
Cicatricial CNV with pigmentary areas

- feeder-vessels that connect the scar to the retinal circulation

**Fig. 27:**
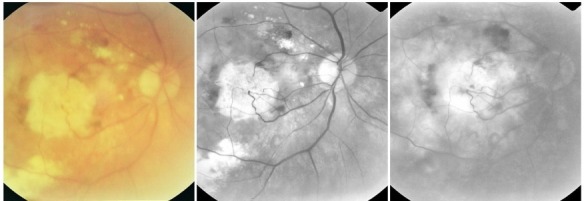
Cicatricial CNV with “feeder-vessel”

- chorioretinal atrophy, that surrounds the cicatricial lesion 

**Fig. 28:**
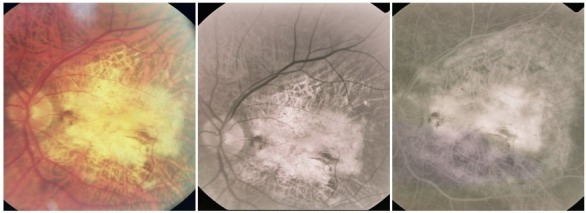
Cicatricial CNV with atrophy around

- chorioretinal folds with distortion of the retinal vessels caused by contraction of the fibrous tissue, sometimes more obvious on FA, where they appear as radial linear hyperfluorescence

**Fig. 29:**
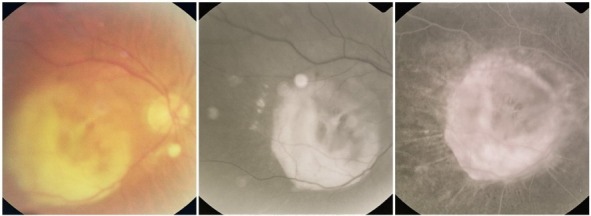
Cicatricial CNV with retinal folds

**9. CNV with massive exudation**

It is a rare form, in which the large exudation extends beyond the temporal vessels and is often associated with subretinal hemorrhage, resulting in a “senile Coats syndrome” aspect and mimicking sometimes a retinal detachment.

**Fig. 30:**
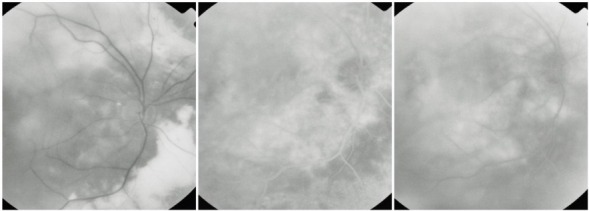
CNV with exudation outside the vascular arcades

**CLASSIFICATION OF ANGIOGRAPHIC ASPECTS IN AMD**

A. Non-exudative AMD

1. RPE defects

2. Hard drusen 

3. Soft drusen 

4. Mixt drusen

5. Cuticular drusen

6. Pseudoreticular drusen

7. Drusen with tendency to confluence

8. Confluent drusen (drusenoid PED)

B. Exudative AMD

1. Classic CNV

2. Occult CNV

- Type 1 occult CNV (fibrovascular PED)

- Type 2 occult CNV ( diffuse exudation)

- Serous or hemorrhagic PED

3. Fibrovascular CNV

4. Cicatricial CNV

5. Retinal angiomatous proliferation

6. Subretinal hemorrhage

7. CNV with RPE tear

8. CNV with massive exudation

## Discussion

FA remains the main diagnostic method for treatable AMD cases. By means of FA, the physician establishes the form and degree of activity of the disease and can evaluate the prognosis. FA can clarify the differential diagnosis in ambiguous cases and can reveal associated lesions. The clinical and angiographic aspects found in AMD have been classified into two main categories depending whether or not exudation was present: non-exudative (“dry”) AMD and exudative (neovascular, “wet”) AMD. The fluorescence angiogram offers information about the extent and the activity of the disease; in cases of neovascular AMD, FA confirms the presence of the choroidal neovascularization and it identifies its characteristics: nature (classic/ occult), activity (exudation), location (extra-, juxta-, subfoveal).

Although initially considered as different forms of the same condition, the multitude of clinical aspects found in AMD are in fact stages of the macular disease, which succeed as the condition progresses. Because of these multiple and various aspects, a standard classification and staging protocol has not been established yet. Many classifications (AREDS, Rotterdam) take into account numerous parameters and are difficult to use in daily clinical practice.

Starting from these considerations, I have tried to elaborate a simplified descriptive classification of the angiographic aspects found in AMD that would also permit a correct staging of the disease and identify the risk factors for progression. 

## References

[1] Pauleikhoff D, Holz FG (1996). Die altersabhangige Makuladegeneration. Epidemiologie, Pathogenese und klinische Differenzierung. Ophthalmologe.

[2] Gass JDM (1973). Drusen and disciform macular detachment and degeneration. Arch Ophthalmol.

[3] Sarks JP, Sarks SH, Killingsworth MC (1994). Evolution of soft drusen in age-related macular degeneration. Eye.

[4] Bird AC, Bressler NM, Bressler SB (1995). An international classification and grading system for age-related maculopathy and age-related macular degeneration. Surv Ophthalmol.

[5] Augood CA, Vingerling JH, de Jong PTVM (2006). Prevalence of age-related maculopathy in older Europeans. The European Eye Study (EUREYE). Arch Ophthalmol.

[6] Gupta OP, Brown GC, Brown MM (2007). Age-related macular degeneration: the costs to society and the patient. Curr Opin Ophthalmol.

[7] Klein R, Klein BE, Jensen SC, Meuer SM (1997). The five-year incidence and progression of age-related maculopathy: the Beaver Dam Eye Study. Ophthalmology.

[8] (1991). Macular Photocoagulation Study Group: Subfoveal neovascular lesions in age-related macular degeneration. Guidelines for evaluation and treatment in the macular photocoagulation study. Arch. Ophthalmol.

[9] Hamada S, Jain S, Sigvagnanavel V, Patel N, Chong NV (2006). Drusen classification in bilateral drusen and fellow eye of exudative age-related macular degeneration. Eye.

[10] Cohen SY, Dubois L, Tadayoni R, Dlhaye-Mazza C, Debibie C, Quentel G (2007). Prevalence of reticular pseudodrusen in age-related macular degeneration with newly diagnosed choroidal neovascularisation. Br J Ophthalmol.

[11] Olsen T, Feng X, Kasper T, Rath P, Steuer E (2004). Fluorescein angiographic lesion type frequency in neovascular age-related macular degeneration. Ophthalmology.

[12] Kertes PJ (2004). Fluorescein angiographic lesion type frequency in neovscular age-related macular degeneration. Evidence-Based Eye Care.

[13] Sarks JP, Sarks SH, Killingsworth MC (1988). Evolution of geographic atrophy of the retinal pigment epithelium. Eye.

[14] Hartnett ME, Weiter JJ, Staurenghi G, Elsner AE (1996). Deep retinal vascular anomalous complexes in advanced age-related macular degeneration. Ophthalmology.

[15] Yannuzzi LA, Negrao S, Iida T (2001). Retinal angiomatous proliferation in age-related macular degeneration. Retina.

[16] Freund KB, Ho IV, Barbazetto IA (2008). Type 3 neovascularisation: the expanded spectrum of retinal angiomatous proliferation. Retina.

[17] Gass JDM (1984). Pathogenesis of tears of the retinal pigment epithelium. Br J Ophthalmol.

[18] Heimann H, Kellner U, Foerster MH (2004). Angiographie-Atlas des Augenhintergrundes.

